# Left common peroneal nerve palsy caused by cross-legged sitting during epidural labor analgesia: a case report

**DOI:** 10.1186/s40981-024-00698-0

**Published:** 2024-02-21

**Authors:** Shunya Ogawa, Hirotsugu Kanda, Hiromichi Kurosaki, Tomoyuki Kawamata

**Affiliations:** Department of Anesthesiology, Wakayama Medical University, Kimiidera 811-1, Wakayama, 641-8509 Japan

**Keywords:** Epidural labor analgesia, Common peroneal nerve palsy, Sitting cross-legged position

## Abstract

**Background:**

Nerve injury in epidural labor analgesia can occur with various potential causes. We report a rare case of left common peroneal nerve palsy after delivery caused by a prolonged period of sitting cross-legged during epidural labor.

**Case report:**

Epidural labor analgesia in a 28-year-old primipara started at 39 weeks of gestation. She sat cross-legged to prompt delivery for approximately 4 h with a break of a few minutes every hour. She had numbness in her left lower limb and difficulty in dorsiflexion of the ankle joint that did not improve until 3 h after delivery. We made a diagnosis of left common peroneal nerve palsy. Most of the symptoms had improved at 2 months postpartum.

**Conclusion:**

Epidural labor analgesia prevented recognition of prolonged peroneal head compression caused by sitting cross-legged. When this position is used to facilitate delivery, it should be released frequently owing to the possibility of a neurologic deficit.

## Background

Paresthesia when giving birth can be caused by prolonged delivery and/or inappropriate positioning. Reported causes of neuropathy associated with delivery include compression of the lumbosacral and sacral plexus nerves and the pudendal nerve by the fetus’s head and pelvic margin and compression of the lateral femoral cutaneous nerve, femoral nerve, pudendal nerve, pudendal nerve, and peroneal nerve by the lithotripsy position and hip flexion during delivery [1, 2]. Nerve injury may be masked when sensory perception is impaired by epidural labor analgesia. This is the first known case report of left common peroneal nerve palsy after delivery caused by sitting for a prolonged period in a cross-legged *agura* position during epidural labor analgesia.

## Case presentation

The patient was 28-year-old Japanese female (height, 163 cm; weight, 65 kg; gravidity, 2 parity: 0) with no complications or notable medical history. Her pregnancy had been uneventful. She requested epidural labor analgesia at 39 weeks of gestation because she felt strong labor pains. A catheter was inserted approximately 5 cm cephalad into the epidural space at the level of L3/4. There was no radiating pain or cerebrospinal fluid reflux during insertion of the epidural catheter. Five minutes after administration of 3 ml of 2% lidocaine with epinephrine, there was no motor paralysis and cold sensation was decreased in the bilateral L1-S areas, and pain during labor was reduced from 9/10 to 2/10 on a numerical rating scale. After a test dose showed a segmental effect, patient-controlled epidural analgesia with 6 ml/h of the combination of 290 ml levobupivacaine 0.1% and 500 μg fentanyl as a continuous background infusion and patient-controlled boluses of 3 ml of the same drug with a lockout interval of 30 min was started. After starting epidural anesthesia, she had slight hypesthesia in both lower limbs due to epidural anesthesia.

Approximately 5 h after installation of the epidural catheter, as a means of prompting delivery, she maintained a cross-legged sitting position with the left leg medial for a total of approximately 4 h with a break of a few minutes every hour. The fetus’s head descended well with straining alone, so there was no suspicion of cephalopelvic imbalance. The baby was delivered about 9 h after insertion of the epidural catheter. The second stage of labor was 49 min, and the third stage of labor was 10 min. The newborn birth length was 50.5 cm and weight was 3319 g. The epidural catheter was removed approximately 30 min after delivery of the baby. At that time, there was no radiating pain or other abnormalities.

The patient was aware that numbness in her left lower limb did not improve 3h after delivery and she had difficulty in dorsiflexion of the ankle joint. Physical examination revealed a positive Tinel’s sign in the left fibular head, numbness and paresthesia in the left lower leg from the lateral side to the dorsal foot, a drop foot, and muscle weakness in a manual muscle test (MMT) of the tibialis anterior muscle at grade 3. There were no findings suggestive of right-sided neurologic deficit, sensory deficit in the left posterior thigh, or motor paralysis of the left tibialis posterior muscle, and we therefore did not suspect spinal cord, plexus, nerve root, or tibial nerve deficits. We diagnosed left common peroneal nerve palsy. The patient was started on vitamin B12 to treat peripheral nerve injury. The MMT grade of the left tibialis anterior muscle had improved to 4 at 4 days after delivery, and she was discharged home on the fifth day after delivery. At the 1 month postpartum examination, MMT of the left tibialis anterior muscle had improved to 5, and at 2 months postpartum, all symptoms had improved except for a very slight sensory insensitivity localized to the left dorsal foot. Written informed consent was obtained from the patient for publication of this case report.

## Discussion

The frequency of neuropathy associated with vaginal delivery varies between 0.3 and 2% [1, 2]. The reported risk factors for postpartum peripheral neuropathy are first delivery, prolonged stage II of labor in the lithotripsy position, and forceps-assisted vaginal delivery [1] and gestational age >41 weeks, late initiation of neuraxial anesthesia, a repeated anesthetic procedure, and newborn birth weight >3.5 kg [3]. In this case, the gestational age was 39 weeks and there was only one anesthetic procedure. The duration of the second stage of this labor, 49 min, was shorter than the median of 1.1 h for nulliparous women with epidural analgesia [4], forceps were not used, and the newborn birthweight was 3.3 kg. The patient was not at high risk of nerve injury in vaginal delivery, with the exception of it being her first delivery.

Neuropathy associated with epidural anesthesia can be caused by neurotoxicity from local anesthetics, nerve injury from epidural puncture, epidural hematoma, and infection. In this case, paresthesia was absent when inserting and removing the epidural catheter. Because there was no right-sided neuropathy and the injury did not span multiple nerve dermatomes, such as the thigh or medial lower leg, we concluded that there was no injury at the spinal cord or plexus level. The absence of weakness of the tibialis posterior muscle and normal sensation in the plantar region and inside of lower leg ruled out a nerve root disorder of L5 or a left tibial nerve neuropathy. The final diagnosis was left common peroneal nerve injury. In this case, the neuropathy had improved approximately 2 months postpartum. Although the prognosis of peripartum nerve injury is generally considered to be good, it should be noted that the median duration of symptoms is 2 months and that neuropathy can persist even after 1 year [1, 2].

**Fig. 1:**
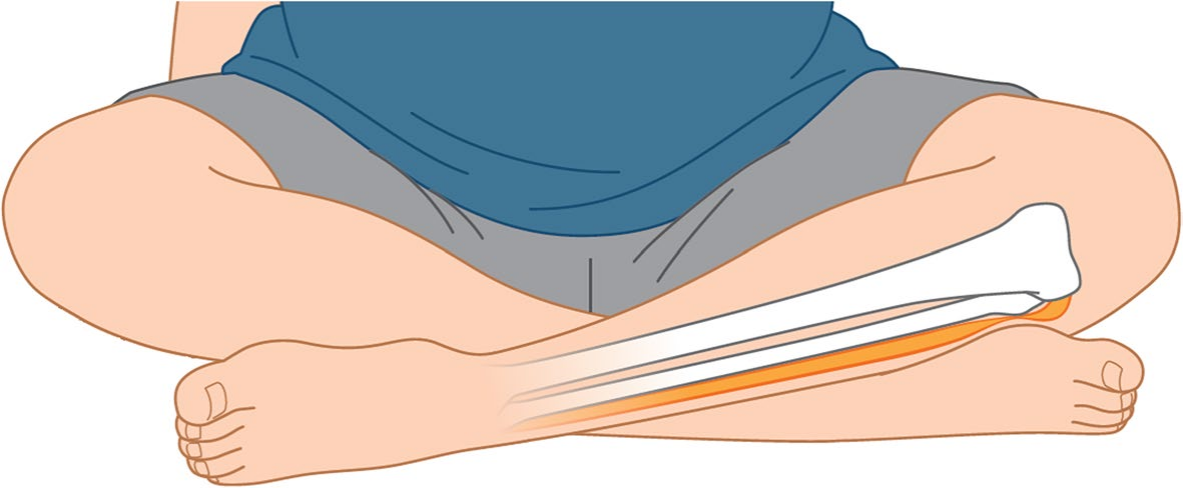
Schema showing compression of the left common peroneal nerve in the peroneal head by the cross-legged sitting position

Yu et al. reported peroneal nerve injuries due to sitting cross-legged for 90 to 240 min in six non-pregnant cases [5]. In our case, the peroneal nerve injury was thought to have been caused by prolonged compression of the left peroneal head by the contralateral leg while the patient was sitting in a cross-legged sitting position to prompt delivery (Fig. 1). Other positions may have caused compression of the peroneal head, but there was no prolongation of the stage II of labor by the lithotripsy position. Common peroneal nerve palsy caused by compression of the peroneal head on the delivery table or by manual compression of the posterior thigh during delivery has been reported [6], but the patient had no clear memory of such manual compression.

Currently, the majority of women give birth either while lying flat or in a semi-sitting position. Standing, sitting, squatting, side lying, and on hands and knees are comparatively uncommon birth positions [7]. Traditional sophrology-based relaxation methods for labor and delivery include yoga exercises such as relaxation in cross-legged and cat postures, muscle relaxation of the abdomen to be used between uterine contractions, and exercises of perineal and perianal muscles [8]. Our patient maintained a cross-legged sitting position as a means of prompting delivery and reducing labor pain [8]. However, the physiologic mechanism of the effect of the cross-legged sitting position during labor remains unclear.

Paralysis of the common peroneal nerve due to compression at the fibular head is a well recognized and relatively common injury among compression nerve injuries [5]. Prolonged cross-legged sitting often leads to a transient numbness or tingling in the top of the foot or the outer part of the lower leg and weakness of the ankles or feet in daily life. Typically, when experiencing numbness, discomfort, or pain in the lower limbs, individuals instinctively or consciously adjust their posture, briefly alleviating compression by altering leg-crossing or adopting a different sitting position to avoid sustained pressure. Due to the diminished sensation in the left leg resulting from lumbar epidural infusion of 0.1% levobupivacaine, the patient likely experienced a reduction or complete absence of the numbness and tingling sensation in the leg and foot, induced by the compression in the vicinity of the fibular head with the *agura* position. Consequently, it is likely that the patient continued to sit in a cross-legged position for an extended period. It is important to recognize that, even with a low concentration of local anesthetic, sensory nerve blockade can lead to compression nerve damage. Therefore, it is necessary for healthcare professionals engaged in labor analgesia to actively encourage patients receiving epidural analgesia to adjust their posture, thereby preventing potential complications.

In conclusion, when a position is used to facilitate delivery with epidural analgesia, the patient should be released from the position frequently to check for neurologic deficits. Further studies are needed to prospectively evaluate the *agura* position during epidural labor, including patient outcomes and satisfaction, the physiologic mechanism, and the frequency of occurrence of nerve injury.

## Data Availability

Not applicable.

## Abbreviation

MMT: Manual muscle test
